# A genome-wide analysis of the *auxin/indole-3-acetic acid* gene family in hexaploid bread wheat (*Triticum aestivum* L.)

**DOI:** 10.3389/fpls.2015.00770

**Published:** 2015-09-30

**Authors:** Linyi Qiao, Xiaojun Zhang, Xiao Han, Lei Zhang, Xin Li, Haixian Zhan, Jian Ma, Peigao Luo, Wenping Zhang, Lei Cui, Xiaoyan Li, Zhijian Chang

**Affiliations:** ^1^Department of Biological Sciences, College of Life Science, Shanxi UniversityTaiyuan, China; ^2^Shanxi Key Laboratory of Crop Genetics and Molecular Improvement, Institute of Crop Science, Shanxi Academy of Agricultural SciencesTaiyuan, China; ^3^Biotechnology Research Insititute, Chinese Academy of Agricultural SciencesBeijing, China; ^4^National Key Facility for Gene Resources and Gene Improvement, Institute of Crop Science, Chinese Academy of Agricultural SciencesBeijing, China; ^5^Triticeae Research Institute, Sichuan Agricultural UniversityChengdu, China; ^6^Department of Biotechnology, College of Agriculture, Sichuan Agricultural UniversityChengdu, China; ^7^Beijing Anzhen Hospital Affiliated to the Capital Medical University/Beijing Institute of Heart Lung and Blood Vessel DiseasesBeijing, China

**Keywords:** bread wheat genome, *Aux/IAA* family, chromosome location, expansion pattern, function prediction

## Abstract

The *Auxin/indole-3-acetic acid* (*Aux/IAA*) gene family plays key roles in the primary auxin-response process and controls a number of important traits in plants. However, the characteristics of the *Aux/IAA* gene family in hexaploid bread wheat (*Triticum aestivum* L.) have long been unknown. In this study, a comprehensive identification of the *Aux/IAA* gene family was performed using the latest draft genome sequence of the bread wheat “Chinese Spring.” Thirty-four *Aux/IAA* genes were identified, 30 of which have duplicated genes on the A, B or D sub-genome, with a total of 84 *Aux/IAA* sequences. These predicted *Aux/IAA* genes were non-randomly distributed in all the wheat chromosomes except for chromosome 2D. The information of wheat Aux/IAA proteins is also described. Based on an analysis of phylogeny, expression and adaptive evolution, we prove that the *Aux/IAA* family in wheat has been replicated twice in the two allopolyploidization events of bread wheat, when the tandem duplication also occurred. The duplicated genes have undergone an evolutionary process of purifying selection, resulting in the high conservation of copy genes among sub-genomes and functional redundancy among several members of the *TaIAA* family. However, functional divergence probably existed in most *TaIAA* members due to the diversity of the functional domain and expression pattern. Our research provides useful information for further research into the function of *Aux/IAA* genes in wheat.

## Introduction

Auxin, the first phytohormone discovered, controls many aspects of plant physiology and morphology including embryogenesis, lateral root initiation, leaf expansion, inflorescence and fruit set (Vanneste and Friml, 2009), and is involved in gene stimulation and regulating the transcription of multiple genes on the molecular level. Several primer auxin-responsive genes have been identified containing the *Aux/IAA, GRETCHEN HAGEN 3* (*GH3*), and *SMALL AUXIN-UP RNA* (*SAUR*) gene families (Abel and Theologis, 1996). With a key role in the auxin signaling pathway, the *Aux/IAA* gene is well known as the transcriptional repressor of the *Auxin Response Factor* (*ARF*) gene family to regulate downstream auxin-regulated genes (Rogg et al., 2001). Moreover, *Aux/IAAs* can mediate the pathway interaction between auxin and light signaling (Halliday et al., 2009) or other hormone signaling such as brassinosteroids (Song et al., 2009), jasmonic acid (Kazan and Manners, 2009), and ethylene (Strader et al., 2010).

The *Aux/IAA* family members encode short-lived nuclear proteins ranging from 18 to 36 kD (Paul et al., 2005). Canonical proteins of the *Aux/IAA* family share four conserved motifs known as domains I–IV. Domain I at the N-terminus contains a leucine repeat (LxLxLx) motif (Tiwari et al., 2001). Domain II is involved in the instability of proteins (Kepinski and Leyser, 2004). Domains III and IV mediate homo-dimerization and hetero-dimerization between the Aux/IAA and ARF proteins via C-terminal dimerization binding sites (Hagen and Guilfoyle, 2002; Tiwari et al., 2004).

Since the initial isolation of *Aux/IAA* family genes in soybean (*Glycine max*) (Walker and Key, 1982), 29 members in *Arabidopsis thaliana* (Paul et al., 2005), 35 members in *Populus trichocarpa* (Kalluri et al., 2007), 26 members in tomato (Wu et al., 2012), sorghum (Wang et al., 2010a), and grape (Birsen et al., 2013), and 31 members in rice (Jain et al., 2006), and maize (Wang et al., 2010b) have been identified. Several *Aux/IAA* family genes control a number of important plant traits. *OsIAA2* enhances the resistance of rice to pathogens (Chen et al., 2009); *OsIAA5* (Peleg et al., 2011), and *OsIAA6* (Jung et al., 2015) are involved in drought tolerance; *SbIAA1* relates to stress response (Wang et al., 2010a); and *VvIAA4* (Birsen et al., 2013), *VvIAA9* (Jung et al., 2014), *SlIAA9* (Wang et al., 2005a; Mazzucato et al., 2015), *SlIAA15* (Deng et al., 2012), and *SlIAA17* (Su et al., 2014) are key regulators of the fruit set process.

Despite extensive studies of *Aux/IAA* in many other plants, little is known about this gene family in bread wheat (*Triticum aestivum* L.), one of the most widely grown crops in the world. Until now, only one *Aux/IAA* gene, *TaIAA1*, was reported to be regulated by epibrassinolide and light (Singla et al., 2006). Bread wheat (AABBDD; 2n = 6x = 42) is a result of hybridization between *T. turgidum* (AABB; 2n = 4x = 28), an allotetraploid originating from a cross of *T. urartu* (AA; 2n = 14) and *Aegilops speltoides* (SS; 2n = 14), and *A. tauschii* (DD; 2n = 14) which occurred approximately 0.43 MYA (million years ago). It is problematic to isolate *Aux/IAA* family genes in bread wheat due to its huge genome that comprises a high scale (>80%) of repetitive sequences (Wicker et al., 2011).

In 2012, the draft genome of bread wheat “Chinese Spring” (“CS”) reading by whole-genome shotgun sequencing was published (Brenchley et al., 2012). Soon afterwards, the sequencing data of *T. urartu* (Ling et al., 2013) and *A. tauschii* (Jia et al., 2013), two progenitors of bread wheat, was released in 2013. These resources have provided a wealth of information about the coding genes of wheat (Saintenac et al., 2013). However, little is known about the distribution and position of these genes on each wheat chromosome and their evolution during the two polyploidization events. Recently, a 17-gigabase draft genome of the bread wheat “CS” produced through sequencing isolated chromosome arms was published (Mayer et al., 2014), making possible the isolation and analysis of gene families on a genomic scale.

In this study, a genome-wide isolation of *Aux/IAA* domains in bread wheat was performed using sequence resources. Eighty-four sequences were isolated and divided into 34 groups based on homology among the A, B and D sub-genomes. Detailed information about wheat *Aux/IAA* genes (*TaIAAs*) was acquired. In addition, the expansion pattern and selection pressure analysis of the *Aux/IAA* family in wheat was deduced.

## Materials and methods

### Searching for the *Aux/IAA* family in wheat, *T. urartu* and *A. tauschii*

The whole-genome sequences of *T. aestivum* was downloaded from the wheat genome URGI database (http://wheat-urgi.versailles.inra.fr/) and Ensembl database (http://plants.ensembl.org). Based on these sequences, a local nucleotide and protein database was established by Basic Local Alignment Search Tool (BLAST, ftp://ftp.ncbi.nlm.nih.gov/blast/executables/blast+/LATEST/). The hidden Markov model (HMM) profile of the *Aux/IAA* family (PF02309) was extracted from the Pfam database (http://pfam.sanger.ac.uk) and the Aux/IAA HMM profile was used to search the local protein database for target hits with the Aux/IAA-domain by HMMER3.0 (http://hmmer.janelia.org/). All non-redundant sequences with expected values lower than 1E-5 were selected and received a conserved domain check using the Pfam tool (http://pfam.xfam.org/) and SMART (http://smart.embl-heidelberg.de/) web server. According to the sequence ID of the Aux/IAA protein in wheat, the coding sequences and genome sequences were isolated from the local nucleotide database. Using the same method, the *Aux/IAA* genes of *T. urartu* and *A. tauschii* were defined from the *T. urartu* genomic database (http://gigadb.org/dataset/100050) and *A. tauschii* genomic database (http://gigadb.org/dataset/100054), respectively.

### Phylogenetic relationships, gene structure, and chromosome location of wheat *Aux/IAA* genes

The exon/intron organization of each *Aux/IAA* gene was illustrated in the Gene Structure Display Server program (Hu et al., 2015) by comparing their coding sequences with genomic sequences. The position of each *Aux/IAA* gene in the wheat chromosomes and the genome sequences of the corresponding wheat chromosomes were determined by BLAST, and the results were displayed using the MapInspect tool (http://mapinspect.software.informer.com/). An unrooted phylogenetic tree for wheat *Aux/IAA* genes was constructed using MEGA 6.0 (Tamura et al., 2013) via the Neighbor-Joining (NJ) method. Duplicated genes in the branch ends of each group belonging to the A, B or D sub-genomes of wheat were regarded as the homologous copies of one *Aux/IAA* gene. All of the *TaIAAs* were named according to their chromosome position and homology among the three wheat genomes.

### Physical and chemical properties, secondary structure prediction, motif display, and phylogenetic analysis for the wheat Aux/IAA proteins

The basic physical and chemical parameters of TaIAA proteins were predicted by the ProtParam tool (http://www.expasy.org/tools/protparam.html). The secondary structures of TaIAAs were analyzed by the net service NPS (https://npsa-prabi.ibcp.fr/cgi-bin/npsa_automat.pl?page=/NPSA/npsa_seccons.html). The conserved domains were investigated using Clustal W (Larkin et al., 2007) by multiple alignment analyses and visualized in Jalview (Waterhouse et al., 2009). Motifs of the TaIAA proteins were displayed with the MEME (Bailey et al., 2009) online tool. MEME found four motifs and the other parameters were defaulted. The phylogenetic analysis of Aux/IAA proteins was performed using the NJ method of MEGA 6.0. The protein sequences of other species such as OsIAAs, AtIAAs, SlIAAs, and VvIAAs were download from the NCBI database (http://www.ncbi.nlm.nih.gov/) according to the Genbank number.

### Expression analysis for *TaIAAs*

The coding sequences of the *TaIAAs* were submitted to the Plex database (http://www.plexdb.org/) to search for corresponding probes. These probes were then used to query the GeneVestigator database (https://genevestigator.com/gv/) to obtain the expression data of *TaIAA* genes in 19 organs of wheat. All transcript data was transformed by log_2_ and the heat map was viewed in the MeV tool (http://www.tm4.org/mev.html).

### Expansion pattern analysis for *TaIAAs*

Segmental replication and tandem duplication are the main repeat methods of the gene family among the lineage-specific expansion. In this study, 84 genomic sequences of *TaIAA*s were investigated for segmental replication and tandem duplication events according to the method established in previous studies (Guo and Qiu, 2013). Paralogs from different sub-genomes of wheat were regarded as segmental replication, while two or more *TaIAA*s situated in the same location of the chromosome were defined as tandem duplication, based on the wheat genome annotation results in the URGI database.

### Collinearity analysis for the *Aux/IAA* family

Phylogenetic trees for the wheat-*T. urartu* and wheat-*A. tauschii Aux/IAA* genes were constructed in MEGA 6.0. Two genes from different species situated in the same branch of the phylogenetic tree were designated orthologs (Koonin, 2005). Based on these orthologous *Aux/IAA* genes, collinearity maps of the *Tu*-wheat A genome and the *Aet*-wheat D genome were output by the genome visualization tool CIRCOS (Krzywinski et al., 2009).

### Ka and Ks calculations

The rate of Ka (non-synonymous substitution rate)/Ks (synonymous substitution rate) was applied to compare the rates of codon evolution in the sub-genome of wheat using barley as an outgroup (Akhunov et al., 2013). The orthologous gene pairs between barley and wheat were used to calculate Ka and Ks in the PAL2NAL server (Suyama et al., 2006) using the codeml program of phylogenetic analysis by maximum likelihood (PAML; Yang, 1997). The barley *Aux/IAA* sequences were downloaded from the PlantTF database (http://planttfdb_v1.cbi.pku.edu.cn:9010/).

## Results

### Searching for *Aux/IAA* genes in wheat

**Figure 1:**
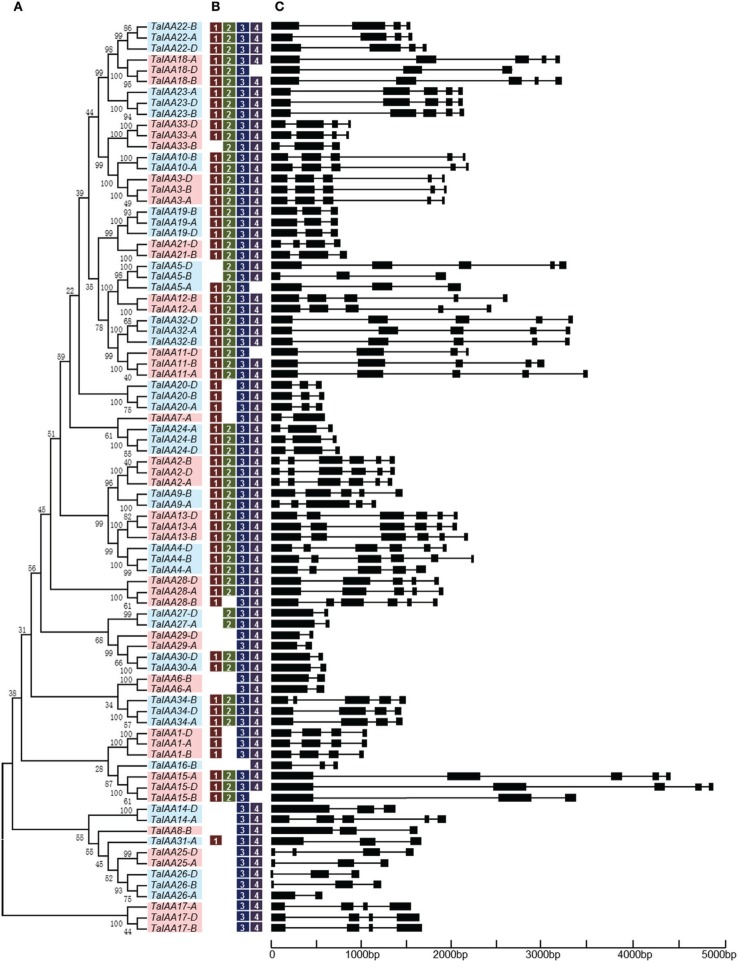
**Phylogenetic relationship, motif and gene structure of wheat ***Aux/IAA*** genes**. **(A)** The phylogenetic tree of *TaIAAs* constructed from a complete alignment of 84 wheat *Aux/IAA* genes using MEGA 6.0 by the neighbor-joining method with 1000 bootstrap replicates. Bootstrap scores are indicated on the nodes and the 34 members of *TaIAA*, most of which contain duplicated genes, are indicated by blue or pink block. **(B)** The conserved motifs of TaIAAs. Motifs were identified by MEME software using the deduced amino-acid sequences of the TaIAAs. The relative position of each identified motif in all TaIAA proteins is shown as 1, 2, 3, and 4, which represented the four conserved domains of IAA. **(C)** Exon/intron structures of *TaIAA* genes. Exons are represented by black boxes and introns by black lines. The sizes of exons and introns can be estimated using the scale below.

A 22.8 GB local database of wheat nucleotide and protein sequences was built. By retrieving wheat protein sequence databases using the *Aux/IAA* HMM file, 127 non-redundant sequences were obtained. Next, 84 full-length protein sequences were detected that contained conserved *Aux/IAA* domains. In addition, corresponding coding sequences and genome sequences were isolated from the local nucleotide database and each *Aux/IAA* gene was located by searching the wheat chromosome genomic sequences using BLASTn (Table 1). An un-rooted tree of the 84 *Aux/IAA* full-length coding sequences was constructed (Figure 1A) to reveal the phylogenetic relationship and all the sequences were divided into 34 groups. Among them, 30 groups have two or three genes from different wheat sub-genomes, and these were regarded as different copies of one member of the *Aux/IAA* gene family.

**Table 1:** *****Aux/IAA*** gene family in wheat**.

**Gene**	**Sequence ID^a^**	**Scaffold**	**Location**	**ORF bp**	**Length AA**
*TaIAA1-A*	TaLoc010775.4	3295285	1AS:31279430-31280500	1071	208
*TaIAA1-B*	Traes_1EEE57162.1	2899111	1BS:2616364-2615360	1042	209
*TaIAA1-D*	Traes_CA4185D11.1	1883869	1DS:70882162-70883423	1079	207
*TaIAA2-A*	Traes_372F80BF9.1	3308105	1AS:163196691-163198564	1349	233
*TaIAA2-B*	Traes_E5A12CB09.1	3449004	1BS:86215073-86216744	1366	235
*TaIAA2-D*	Traes_565BF8DDA.1	1909974	1DS:52657909-52659817	1367	236
*TaIAA3-A*	Traes_EADB6A2A0.1	3311117	1AS:166600171-166602694	1936	199
*TaIAA3-B*	Traes_8A19C460B.1	3479210	1BS:113674501-113677207	1964	199
*TaIAA3-D*	Traes_0898FA765.1	92692	1DS:64906433-64908913	1932	199
*TaIAA4-A*	TaLoc011447.4	3922404	1AL:216593028-216593434	1731	301
*TaIAA4-B*	Traes_BFB6F2ABC.1	1095310	1BL:232504409-232504535	2254	292
*TaIAA4-D*	TaLoc007655.5	2239906	1DL:119721561-119717330	1954	277
*TaIAA5-A*	Traes_859346448.1	3893649	1AL:235709512-235704959	2732	242
*TaIAA5-B*	Traes_4B1CFEF1C.1	3916489	1BL:278915207-278919038	1959	231
*TaIAA5-D*	Traes_4F703A523.1	2279082	1DL:125510254-125514512	3289	286
*TaIAA6-A*	Traes_8F14EF2CE.1	6390277	2AL:237394204-237395143	598	168
*TaIAA6-B*	Traes_E592F41F5.1	8087827	2BL:325907516-325908479	590	168
*TaIAA7-A*	Traes_A598DE96B.1	6368366	2AL:253631713-253642960	600	159
*TaIAA8-B*	Traes_B2D711406	8005976	2BL:332291714-332296345	1642	328
*TaIAA9-A*	Traes_771897131.1	3339447	3AS:19556131-19557111	1168	263
*TaIAA9-B*	Traes_99D28E887.1	10603561	3B:113552214-113552559	1480	242
*TaIAA10-A*	Traes_B855A6F86.1	3407523	3AS:22626398-22625699	2194	219
*TaIAA10-B*	Traes_EC3C8E7C1.1	10445740	3B:133879562-133881697	2162	203
*TaIAA11-A*	Traes_650C7A7A9.1	3299314	3AS:47736317-47738563	3529	266
*TaIAA11-B*	Traes_F684DCAB2.1	10498911	3B:181982813-181985970	3041	279
*TaIAA11-D*	Traes_BA5B9A06A.1	1300831	3DS:19461793-19465907	2195	230
*TaIAA12-A*	Traes_C8CC634C4.1	36155	3AL:151818719-151826972	2437	262
*TaIAA12-B*	Traes_0D5F4D67A.1	10344697	3B:488692481-488695888	2621	257
*TaIAA13-A*	Traes_E77F7C3EE.1	4414529	3AL:135517081-135520321	2062	342
*TaIAA13-B*	Traes_F880EDE81.1	10486611	3B:493606171-493606667	2188	344
*TaIAA13-D*	Traes_EC1DE49E1.1	6954745	3DL:38786327-38788145	2068	326
*TaIAA14-A*	Traes_7A2CED8E7.1	4263764	3AL:133077764-133073796	1981	248
*TaIAA14-D*	Traes_0BAE401F0.1	6949978	3DL:106433431-106435603	1387	333
*TaIAA15-A*	Traes_58F89633A.1	7155902	4AL:74099030-74104019	4463	362
*TaIAA15-B*	Traes_74A9C6245.1	4491507	4BS:24412418-24416296	3422	328
*TaIAA15-D*	Traes_BE7FDCA21.1	2321248	4DL:59775415-59780351	4937	361
*TaIAA16-B*	Traes_8A04B545C.1	4913821	4BL:158664312-158665608	739	104
*TaIAA17-A*	Traes_CD68C12EF.1	7150674	4AL:130935545-130937270	1582	201
*TaIAA17-B*	Traes_92DD5D26B.1	4882572	4BL:198119295-198121461	1664	209
*TaIAA17-D*	Traes_03ABB2A80.1	2305688	4DL:52397663-52399704	1657	204
*TaIAA18-A*	Traes_7C1851556.1	1535009	5AS:76737098-76736271	3227	254
*TaIAA18-B*	Traes_940DB64ED.1	2245740	5BS:77593270-77597121	3206	270
*TaIAA18-D*	Traes_76A4D5D4E.1	324017	5DS:30146329-30150135	2703	221
*TaIAA19-A*	Traes_B39049539.1	1552256	5AS:60585303-60590018	741	187
*TaIAA19-B*	Traes_8CD712BC5.1	2267405	5BS:101843501-101844847	742	198
*TaIAA19-D*	Traes_66E5D2F6D.1	273208	5DS:37692357-37693629	744	187
*TaIAA20-A*	Traes_A878DC43C.1	2806306	5AL:82165282-82165540	588	134
*TaIAA20-B*	Traes_BFE717C47.1	10860770	5BL:199348871-199350113	596	134
*TaIAA20-D*	Traes_E62BE613E	4545184	5DL:114545445-114546480	584	134
*TaIAA21-B*	Traes_9301BD154.1	10732661	5BL:215297647-215299043	835	213
*TaIAA21-D*	Traes_C2F7D9273.1	4537915	5DL:125470184-125470754	769	160
*TaIAA22-A*	Traes_F845558C3.1	2770845	5AL:105778316-105779350	1157	215
*TaIAA22-B*	Traes_EC006AD0C.1	10845556	5BL:218543827-218546250	1571	254
*TaIAA22-D*	Traes_A9B098305.1	4496111	5DL:127979413-127980640	1759	253
*TaIAA23-A*	Traes_AB296E85D.1	2681049	5AL:107424300-107426935	2136	231
*TaIAA23-B*	Traes_C86AC392A.1	5138938	5BL:216239503-216242516	2165	240
*TaIAA23-D*	Traes_1CFC72F63.1	4490955	5DL:129481978-129485104	2148	233
*TaIAA24-A*	Traes_B7F55FB76.1	2770845	5AL:124461002-124461327	692	161
*TaIAA24-B*	Traes_67E73FC97.1	10921140	5BL:241395575-241397123	743	178
*TaIAA24-D*	Traes_5889C544A.1	4490001	5DL:139990355-139991795	774	182
*TaIAA25-A*	Traes_DEE1DEE94.1	4395378	6AS:31608615-31611001	1557	117
*TaIAA25-D*	Traes_E0F641998.1	2125475	6DS:21381308-21382341	1597	126
*TaIAA26-A*	Traes_0A9F83AC3.1	4403562	6AS:35897029-35897839	582	123
*TaIAA26-B*	Traes_89A33174B.1	2993958	6BS:3056991-3057802	1228	99
*TaIAA26-D*	Traes_F8829CE69	2057257	6DS:15111844-15113547	980	101
*TaIAA27-A*	TaLoc012813.1	5808478	6AL:200726057-200726703	647	176
*TaIAA27-D*	Traes_24EA20B3C.1	1744075	6DL:167286203-167286913	640	173
*TaIAA28-A*	Traes_47CDA3A42.1	5742908	6AL:205873664-205877424	1922	284
*TaIAA28-B*	Traes_C9E1E6D09.1	4318404	6BL:202673596-202673672	1858	295
*TaIAA28-D*	Traes_AF62080FF.1	2949722	6DL:172254615-172247541	1865	283
*TaIAA29-A*	Traes_AA598441C.1	960096	7AS:20831150-20831527	456	120
*TaIAA29-D*	Traes_864667028.1	3901523	7DS:25442317-25442701	469	122
*TaIAA30-A*	Traes_D88B8D286.1	4201216	7AS:61921151-61921477	614	172
*TaIAA30-D*	TaLoc011132.1	3901524	7DS:25906078-25906449	577	161
*TaIAA31-A*	Traes_C645A238F.1	4069273	7AS:100746802-100748856	1694	229
*TaIAA32-A*	Traes_354EEE44E.1	4508087	7AL:107594908-107598785	3320	240
*TaIAA32-B*	Traes_74071485F.1	6706664	7BL:190772370-190773270	3324	236
*TaIAA32-D*	Traes_3AB665CC2.1	3345200	7DL:185819441-185820097	3349	241
*TaIAA33-A*	Traes_4878FA904.1	4552230	7AL:118339820-118341247	863	207
*TaIAA33-B*	TaLoc020878.2	6738232	7BL:189490619-189490875	761	164
*TaIAA33-D*	Traes_01578227E.1	3377355	7DL:166166562-166168131	888	181
*TaIAA34-A*	Traes_920F5EB57.1	4547012	7AL:154013235-154012654	1475	252
*TaIAA34-B*	Traes_20B08C649.1	6746741	7BL:218108685-218108782	1498	245
*TaIAA34-D*	Traes_36F56E8E0.1	3394907	7DL:177360424-177365747	1442	253

a *The sequence name beginning with TaLoc and Traes means downloaded from the wheat genome URGI database and Ensembl database, respectively*.

### Genome distribution of wheat *Aux/IAA* genes

**Figure 2:**
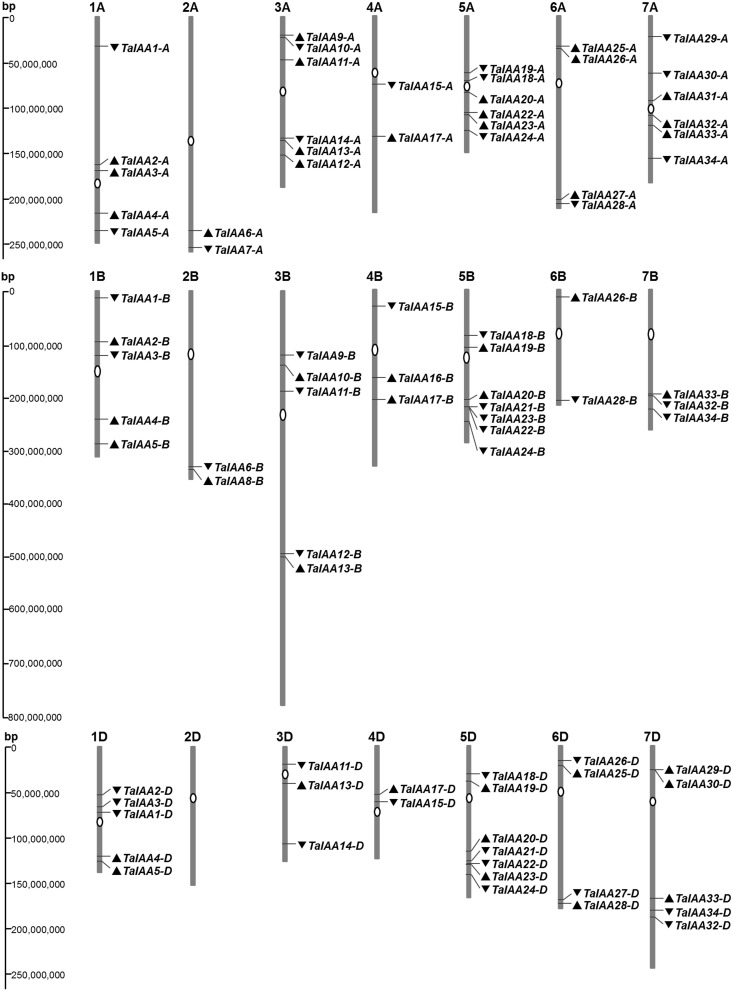
**Chromosome distribution of ***TaIAA*** family in wheat**. White ovals on the chromosomes (vertical bar) indicate the position of centromeres. The arrows next to gene names show the direction of transcription. The position of each gene can be estimated using the left scale. The chromosome numbers are indicated at the top of each bar.

The genome distribution of 84 wheat *Aux/IAA* genes is shown in Figure 2. Respectively, 31, 27, and 26 *Aux/IAA* genes are non-randomly distributed in the three wheat sub-genomes. According to chromosome position and genomic homology, all these wheat *Aux/IAA* genes (*TaIAAs*) were named *TaIAA1-A*~*TaIAA34-D* and distributed on every wheat chromosome except for chromosome 2D (Figure 2). Each of the 20 *TaIAA* genes (*TaIAA1, 2, 3, 4, 5, 11, 13, 15, 17, 18, 19, 20, 22, 23, 24, 26, 28, 32, 33*, and *34*) contains three copies in chromosome A, B and D; 10 *TaIAA*s have *two* copies each including *TaIAA-A*/*-B* (*TaIAA6, 9, 10*, and *12*), *TaIAA-B*/*-D* (*TaIAA21*), and *TaIAA-A*/*-D* (*TaIAA14, 25, 27, 29* and *30*); and *TaIAA7, 8, 16*, and *31* have just one copy in wheat chromosomes.

**Figure 3:**
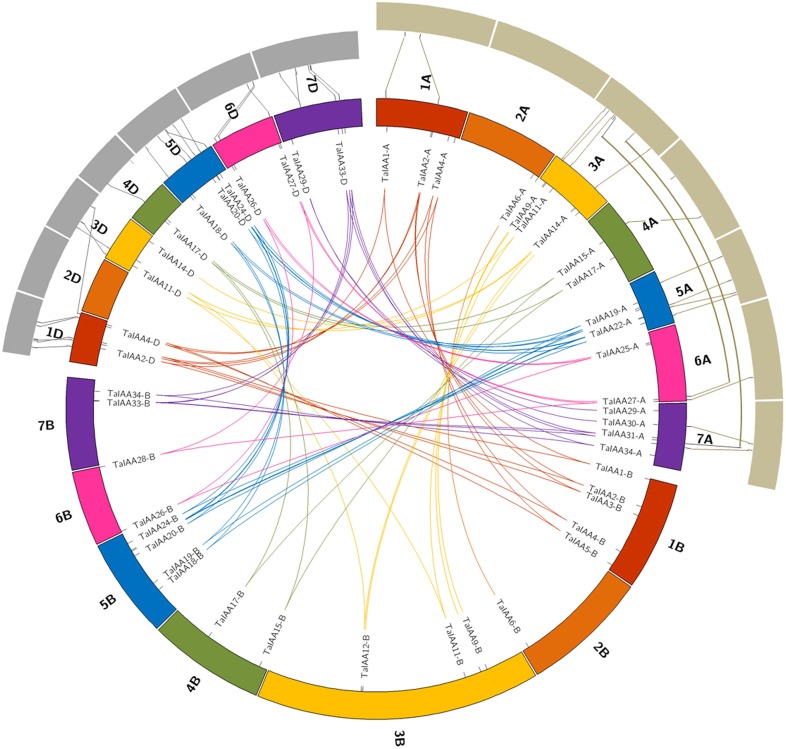
**Duplicated ***TaIAA*** genes of wheat homologous groups and the collinearity among ***TaIAAs, TuIAAs***, and ***AetIAAs*****. Seven homologous groups of wheat chromosomes are displayed in different colors. Duplicated genes of each homo-group are linked using lines with corresponding color. The gray annulus on the periphery represents chromosomes of *T. urartu* and *A. tauschii*. The collinearity among *TaIAAs, TuIAAs*, and *AetIAAs* were signified by the lines between the orthologous gene-pairs.

There is a high homology among the scaffolds of one *TaIAA* member which belongs to the A, B or D sub-genome (Figure 3), proving that the *TaIAA* genes experienced two segmental replication events in wheat except for the four single-copy *TaIAA* genes. However, it could also be the case that the segmental replication genes of the four *TaIAA*s were lost after the expansion event. Furthermore, three genomic loci containing two *TaIAA* genes each were defined in the A sub-genome, and the B or D sub-genomes have four such loci (Supplementary Table 1), implying that tandem duplication is also an expansion pattern of the *TaIAA* gene family.

### Gene structure of wheat *Aux/IAA* genes

Schematics of gene structures generated by the GSDS utility are shown in Figure 1C. In the *TaIAA* gene family, the number of introns ranges from 1 to 5. Among the 30 *TaIAAs* which contains two or three copies, 17 *TaIAA* genes (*TaIAA1, 2, 3, 6, 10, 12, 13, 17, 19, 20, 22, 23, 24, 27, 29, 30*, and *32*) have the same gene structures, and the remaining 13 have only one or two differences in the number of introns in each group. Overall, a highly similar gene structure is exhibited in the same phylogenetic cluster of the *TaIAA* genes, suggesting that duplicated genes may have the same function.

### Characteristics of TaIAA protein sequences

Some physical and chemical properties of wheat Aux/IAA proteins are shown in Supplementary Table 2. The predicted molecular mass varies from 21.9 kD for TaIAA1-A to 37.9 kD for TaIAA15-A amongst the Aux/IAA proteins. The negative grand average of the hydropathicity (GRAVY) index showed that all wheat Aux/IAA polypeptides are hydrophilic except TaIAA26-A, suggesting that they are more likely nucleoproteins than membrane proteins. In addition, 69 out of 84 (82.1%) wheat Aux/IAA proteins possess a stability index of more than 40 and might be unstable *in vitro*.

**Figure 4:**
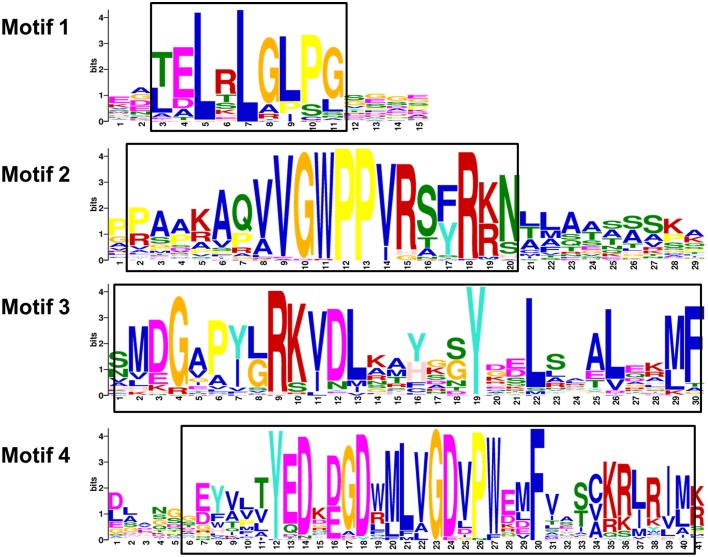
**Motifs of wheat Aux/IAA proteins**. The sequence logos are based on multiple alignment analysis of 84 wheat Aux/IAA proteins performed with Clustal W. The bit score indicates the information content for each position in the sequence. Positions of conserved domains are boxed.

The results of multiple alignment and motif distribution analyses of TaIAA proteins showed that 48 out of 84 (57.1%) wheat Aux/IAA proteins contain the same domains, known as domains I, II, III and IV (Supplementary Figure 1). Motifs 1, 2, 3, and 4 are located in these four domains, respectively (Figures 1B, 4). Eighteen (21.4%) wheat Aux/IAA proteins missed one domain (I, II, or IV), while 17 (20.2%) proteins missed domains I & II; just one (1.2%) protein, TaIAA16-B, lacked three domains (I & II & III). Generally, TaIAA proteins within the same phylogenetic group have similar domains and motifs. Domain I (Motif 1) contains the LxLxLx motif, a typical leucine-rich region that was shown in most TaIAA proteins, which has been shown to act as a strong transcriptional repressor (Tiwari et al., 2004). Domain II contains VGWPP, the core sequence of the target site for wheat Aux/IAA protein degradation. Dominant mutation in this region causes Aux/IAA proteins to fail to resolve via the ubiquitin pathway (Kepinski and Leyser, 2005). Domains III and IV are comparatively more conserved. A β*αα* structure existing in domain III (Motif 3) appeared among all the TaIAA proteins except TaIAA16-B (Figure 1B). It was found that this fold plays an important role in the dimerization of Aux/IAA proteins. Most of the wheat Aux/IAA proteins include two hypothetical nuclear localization signals (NLS). The first bipartite NLS is comprised of two elements: one between domains I and II, and the other in domain II (Supplementary Figure 1). The second element, the SV40-type NLS, is located in domain IV (Supplementary Figure 1). The possible function of these NLSs may be to transfer TaIAA proteins into the nucleus. Interestingly, the conserved residue in the bipartite NLS is KP in TaIAA proteins, while it is KR in rice Aux/IAA proteins (Jain et al., 2006). Therefore, further study into the bipartite NLS in wheat is required. In addition, the existence of phosphorylation sites in several TaIAA proteins partly indicates that these proteins can be extrapolated as short-lived proteins (Supplementary Figure 1).

### Phylogenetic analysis of TaIAA proteins

The phylogenetic tree was built with the sequences of 159 Aux/IAA proteins, including 84 TaIAAs, 31 OsIAAs, 29 AtIAAs, and 15 Aux/IAA proteins with known functions from other species. The information of all the above sequences is listed in Supplementary Table 3.

**Figure 5:**
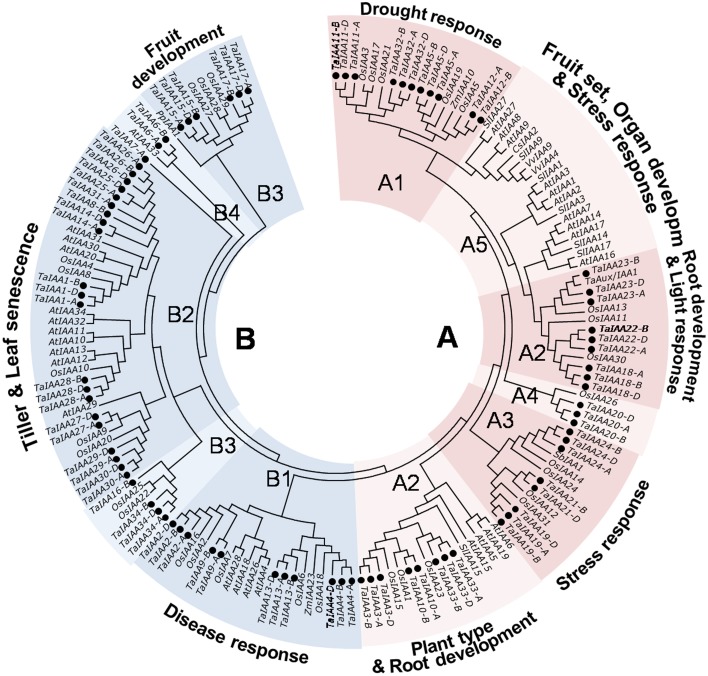
**Phylogenetic relationship of Aux/IAA proteins among wheat and another species**. The full-length amino-acid sequences of 85 wheat, 31 rice, 29 *Arabidopsis*, 7 tomato, 2 maize, 2 grape, 1 sorghum, and 1 pear genes were aligned by Clustal W and the phylogenetic tree was constructed using MEGA 6.0 by the neighbor-joining method with 1000 bootstrap replicates. Each TaIAA protein is indicated by a black dot. Two major groups, group A and B, are represented by the red and blue. The functions of some clades were annotated.

The phylogenetic tree shows that TaIAA proteins can be classified into two major groups: A and B (Figure 5). Based on the clustering tree of OsIAAs (Jain et al., 2006), groups A and B can be further separated into several subgroups: A1, A3, and A4 contain monocotyledon IAA proteins; A5 contains dicotyledon proteins; and A2, B1, B2, B3, and B4 are all types of IAA protein. Moreover, monocot and dicot IAA proteins are not gathered for one class in every subgroup, while the paralogous proteins of each TaIAA are clustered to the same branch, similar to the clustering result in Figure 1A. Orthologous genes usually have similar biological functions (Li et al., 2005). In group A1, OsIAA5 was induced by drought (Peleg et al., 2011), suggesting that TaIAA12 may be related to drought resistance. In addition, OsIAA2 and OsIAA6 in group B1 were induced by pathogens (Chen et al., 2009), OsIAA1 in group A2 regulates plant type (Song et al., 2009), SbIAA1 in group A3 may be related to stress response (Wang et al., 2010a), and OsIAA4 in group B2 regulates plant tiller (Song and Xu, 2013), implying that their orthologs, such as TaIAA1, 9, 10, 13, and 24, may have similar functions in wheat.

### Expression pattern of *TaIAAs* in wheat

**Figure 6:**
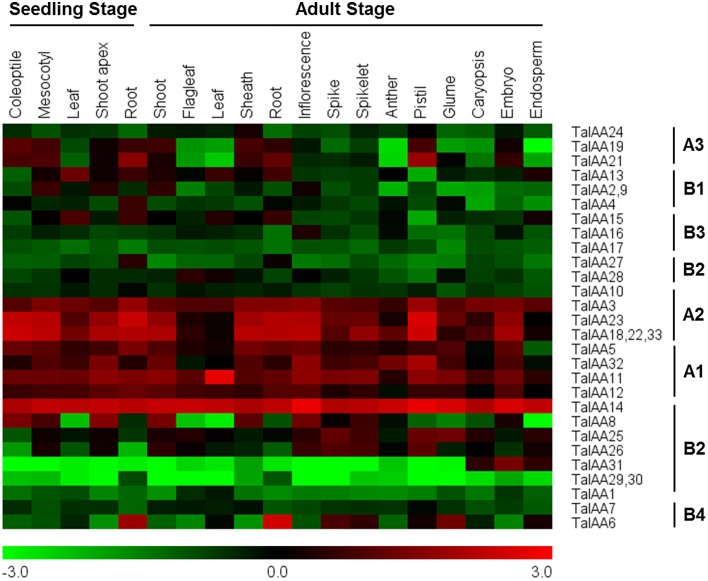
**Heatmap of expression profiles for ***TaIAA*** genes across different organs of seedling and adult stages**. The expression data were generated from GeneVestigator database and viewed in MeV software. The relative expression level of a particular gene in each row was normalized against the mean value by log_2_ transformation. The color scale below represents expression values, green indicating low levels and red indicating high levels of transcript abundance.

The coding sequences of 34 *TaIAAs* were used to search the Plex database for corresponding probes. All gene copies of each *TaIAA* gene share one probe. The probes of *TaIAA20* and *TaIAA34* were not found. Several genes have the same probe, including *TaIAA2, 9*; *TaIAA18, 22, 33*; and *TaIAA29, 30*. Finally, 28 probes for 32 *TaIAAs* were obtained from the GeneVestigator database (Supplementary Table 4). The expression profile of 32 *TaIAAs* in 19 wheat organs covering the seedling to adult stage is shown as a heat map (Figure 6). *TaIAAs* were divided into eight classes according to the subgroups of phylogenetic analysis. In general, the majority of genes in groups A3, B1, B2, and B3 are expressed in vegetative organs of wheat, and genes in group A3 are also expressed in the pistil and embryo. Moreover, all genes in A1, A2 and *TaIAA14* of B2 have high expression levels in the vast majority of wheat organs throughout the entire growing stage. In addition, *TaIAA6* and *TaIAA27* are specifically expressed in the roots, while *TaIAA16, TaIAA28*, and *TaIAA31* are specifically expressed in inflorescence, flag leaf and seed, respectively. *TaIAA1, 7, 10, 17, 29*, and *30* showed low expression in wheat.

### Synchronic analyses of *Aux/IAA* families among *T. urartu, A. tauschii*, and wheat

Twenty-seven *TuIAA*, 31 *TaIAA-A*, 28 *AetIAA*, and 26 *TaIAA-D* sequences were used to construct the phylogenetic tree, and pairs of 22 and 21 *Ta-A*/*Tu* and *Ta-D*/*Aet* orthologs were identified (Supplementary Figure 2). The synchronic analysis results of 16 pairs (just 16 of the 27 *TuIAA* sequences have chromosome location information (Supplementary Table 5) of *TuIAA* and *TaIAA-A* showed that there are 14 (87.5%) homologous genes located on the same chromosome, including 1A, 3A, 4A, 5A, and 6A, while 20 of the 21 pairs (95.2%) between *AetIAA* and *TaIAA-D* are located on 1D, 3D, 4D, 5D, 6D, and 7D (Figure 3). In general, there is a good collinearity in *Aux/IAA* families among *T. urartu, A. tauschii*, and wheat, which suggests that the evolution of the *Aux/IAA* family has been conservative following the formation of hexaploid wheat. However, *TaIAA27-A* and *TaIAA32-A* on wheat chromosomes 6AL and 7AL, respectively correspond to chromosome 3A of *T. urartu*, and *TaIAA5-D* on wheat chromosome 1DL corresponds to chromosome 3DL in *A. tauschii*. Furthermore, a difference in chromosome location caused by pericentric also exists in several orthologous gene pairs, indicating that chromosomal inversion and crossover have occurred in the evolutionary process of the *Aux/IAA* family in wheat.

### Adaptive evolution analysis of the *TaIAA* family

To determine which type of Darwinian selection decided the process of gene divergence after duplication, the Ka/Ks substitution ratio was applied to the coding sequences of 12 pairs of orthologs between barley and wheat *Aux/IAA* family (Supplementary Figure 3). In general, Ka/Ks ratio > 1 means positive selection, ratio < 1 means purifying selection and ratio = 1 means neutral evolution (Akhunov et al., 2013). The Ka/Ks ratios were always less than 1 for *TaIAAs* and ranged from 0.0023 to 0.5444 (Table 2), suggesting that the *Aux/IAA* family has undergone purifying selection in wheat.

**Table 2:** **Ka/Ks ratio of the duplicated ***Aux/IAA*** genes in wheat using barley as an outgroup**.

**Gene**	**A-genome**	**B-genome**	**D-genome**
*TaIAA3*	0.1181	0.1084	0.1642
*TaIAA5*	0.0805	0.3479	0.3058
*TaIAA9*	0.4163	0.3521	–
*TaIAA11*	0.4350	0.5444	0.3777
*TaIAA12*	0.2286	0.2751	–
*TaIAA13*	0.4020	0.4177	0.4284
*TaIAA18*	0.0887	0.1156	0.1076
*TaIAA19*	0.2721	0.2659	0.2772
*TaIAA22*	0.0023	0.1478	0.1437
*TaIAA23*	0.0620	0.0456	0.0374
*TaIAA28*	0.1392	0.0878	0.1331
*TaIAA32*	0.2021	0.2560	0.2511

## Discussion

### Genome-wide isolation of gene families in hexaploid bread wheat

Sequencing projects provide an opportunity for the isolation of gene families in a genome-wide scan. However, it is a great challenge for analysis of polyploid genomes because the relatedness of homeologous sub-genome sequences makes it difficult to assign isolated sequences to the specific chromosome from which they are derived. Until now, some gene families, such as *WRKY* genes (Okay et al., 2014) and nucleotide-binding site (NBS) domain-containing genes (Bouktila et al., 2015), were isolated in wheat. Yet the position of these family genes on wheat genomes and their homologous relationship was still unknown, which leads to the studies on functional divergence and redundancy of duplicated genes could not be proceeded. So compared with rice or maize, the genomic research into hexaploid wheat has been slow for a long time in crops. With the benefit of the new bread wheat draft genome recently through sequencing isolated chromosome arms, we isolated the *Aux/IAA* gene family in wheat. Unlike previous studies, all the *TaIAA* genes were mapped in homoeologous chromosome groups and sequences in the same group of the phylogenetic tree were considered the duplicated genes of one *TaIAA* member. Based on this perspective, the following analysis reflects some special duplicable and functional characteristics of the *Aux/IAA* gene family in wheat.

### Expansion and the fate of duplicated *Aux/IAA* family genes in wheat

In this study, we isolated 27 and 28 *Aux/IAA* genes from the genomes of *T. urartu* and *A. tauschii*, respectively, which are similar in their number of *Aux/IAA* family genes with tomato (26), sorghum (26), *A. thaliana* (29), rice (31), and maize (31). We deduce that the allopolyploidization event that crossed *T. urartu* and *A. speltoides* resulted in the *Aux/IAA* family being replicated in *T. turgidum*. This tetraploid emmer wheat subsequently hybridized with *A. tauschii*, resulting in the second replication of the *Aux/IAA* family in hexaploid bread wheat. The tandem duplication also occurred via this process, eventually forming the 84 wheat *Aux/IAA* sequences including 31, 27, and 26 genes from the A, B, and D sub-genomes of wheat, respectively. A good collinearity of *Aux/IAAs* was shown between wheat and the A and D genome donors *T. urartu* and *A. tauschii* (Figure 3).

After the expansion of *Aux/IAAs* in wheat, the duplicated genes underwent an evolutionary process of purifying selection that can be inferred by their Ka/Ks ratios (Table 2). Therefore, the duplicated genes from the A, B, or D sub-genomes of wheat have a high conservation and share one probe in the expression database (Figure 6). However, the inversion and crossover also happened in wheat chromosomes during the evolutionary process, causing the different fates of several duplicated *Aux/IAA* genes. One such fate was the loss of duplicated genes such as *TaIAA14-B* or *TaIAA17-B* (Figure 1). Moreover, the positions of some duplicated genes changed, involving not only the shift of linear array location and transcription direction (Figure 2), but also the exchange of position between two chromosomes. Based on the collinearity of *Aux/IAA* genes between *T. urartu* and wheat, we infer that *TaIAA27* and *TaIAA32* were originally on chromosome 3A and then transferred to chromosome 6A and 7A, respectively by interchromosomal translocation (Figure 3). The translocation phenomenon has appeared in previous research (Salse et al., 2008; Ma et al., 2013). In addition, the other important peculiar feature of duplicated *Aux/IAA* genes is the altering of the conserved motif, including degenetation and neofunctionalization according to Yang's view (Yang et al., 2006). For example, *TaIAA11-A, TaIAA11-B*, the orthologs of the D subgenome and AEGTA27928 all contain the four motifs of the *Aux/IAA* family, while *TaIAA11-D* lacks motif 4. Other duplicated genes obtained a new motif. Compared with *TaIAA5-A*, the motifs of *TaIAA5-B* and *TaIAA5-D* transform Motif 1 + 2 + 3 to Motif 2 + 3 + 4, which may bring out a novel function.

### Functional divergence of *TaIAAs*

Based on the phylogenetic tree of *Aux/IAA* proteins (Figure 5), we infer that the *Aux/IAA* genes divided before the split of monocots and dicots (90 MYA). Later, the second differentiation happened in a lineage-specific manner in the rice lineage and *Triticeae* before the divergence of *Triticum* and *Aegilops* (3~40 MYA). Therefore, the wheat *Aux/IAA* family has experienced at least two changes.

Despite several genes, including *TaIAA29* and *TaIAA30*, that may have a functional redundancy because of their identical expression patterns (Figure 6), the functions of most *TaIAAs* are probably diverse due to the diversity of the functional domain and expression pattern.

In this study, *TaIAA27* did not contain domain I and the protein may function differently to the classical IAA repressors. Reference on the research in *Arabidopsis* (Dreher et al., 2006), absent of Domain II in TaIAA proteins may lead to enhanced auxin responses by blocking the degradation of IAA proteins, such as *TaIAA14*. However, *TaIAA1* and *TaIAA7* present a very low expression level in various organs throughout the growing period of wheat, suggesting that the degradation of these proteins were independent on domain II- mediate auxin responses. Moreover, *TaIAA20*, which also lacks domain II, has not had any expression data detected at all, implying that *TaIAA20* might be a pseudogene like some *AtIAAs* (Reed, 2001). In addition, *TaIAA34* similarly has no expression data but it does have all four domains, which implies that TaIAA34 is expressed through a particular pattern according to similar research into *OsIAAs* (Jain et al., 2006) and *ZmIAAs* (Wang et al., 2010b).

The genes in groups A1 and A2 of the phylogenetic tree exercise an important function in terms of root development and responding to the environment. Some *TaIAAs* in these two groups are highly expressed in various organs and involved in almost the entire growth process of wheat. Among them, *TaIAA11* from group A1 is highly expressed in leaves, as well as its orthologous *OsIAA3* (Nakamura et al., 2006), which shows that *TaIAA11* play a key role in leaf development. *TaIAA23* from group A2 shows higher expression levels in leaves and roots than in other wheat organs, and *TaIAA23-B* shows a sequence similarity as high as 95.3% with *TaAux/IAA1* (AJ575098) which is sensitive to light and induced by auxin and brassinosteroids (Singla et al., 2006). In addition, *TaIAA27* and *TaIAA31* exhibit tissue-specific expression in roots and seeds, respectively.

## Conclusion

Bread wheat is an important worldwide crop with a huge genome and highly repetitive transposable elements. In summary, 34 *Aux/IAA* genes including 84 duplicated genes in total were isolated from the wheat genome and located in 41 wheat chromosomes (except chromosome 2D). The *TaIAA* family has been replicated twice in the two allopolyploidization events of bread wheat, when the tandem duplication also occurred. The duplicated genes have undergone an evolutionary process of purifying selection, resulting in the high conservation of copy genes among the sub-genomes and functional redundancy among several members of the *TaIAA* family. However, functional divergence probably existed in most *TaIAA* members due to the diversity of the functional domain and expression pattern.

## Author contributions

Conceived and designed the experiments: XyL, ZC, LQ. Performed the experiments: LQ, XZ, HZ, XL, WZ, LC, PL. Analyzed the data: LQ. Contributed reagents/materials/analysis tools: XyL, LQ, JM, XH. Wrote the manuscript: LQ, LZ, ZC.

## Funding

This study is funded by National Natural Science Foundation of China (31171839), Shanxi Province Science Foundation for Youths (2015021145), Technologies R & D Program of the Shanxi Academy of Agricultural Sciences (15YGG01), The Key Program of the Shanxi Academy of Agricultural Sciences (YZD1501), Shanxi Province Technologies R & D Progaram (20150311001-1), Shanxi Province S & T Infrastructure Development Program (2015012001-13), and Shanxi Province finance supported agricultural projects (2014ZYFZ-03).

### Conflict of interest statement

The Associate editor, Dr Tiegang Lu, declares that despite being affiliated with the same institution as the co-author Dr Xiao Han, the review process was handled objectively. The authors declare that the research was conducted in the absence of any commercial or financial relationships that could be construed as a potential conflict of interest.
