# Supplementary material for: A genome-wide analysis of the auxin/indole-3-acetic acid gene family in hexaploid bread wheat (Triticum aestivum L.)
Source: Front Plant Sci. 2015 Sep 30;6:770. doi: 10.3389/fpls.2015.00770 (PMC4588698; doi:10.3389/fpls.2015.00770)
Supplement: Supplementary file 1 [file Table1.DOCX]

***Supplementary Material***

**Genome-wide analysis of the auxin/indole-3-acetic acid gene family in hexaploid bread wheat (*Triticum aestivum* L.) reveals specific duplicable and functional characteristics**

**Linyi Qiao, Xiaoyan Li*, Zhijian Chang***

*** Correspondence:**

Zhijian Chang: [czjsxaas@126.com](mailto:czjsxaas@126.com);

Xiaoyan Li: [xiaoyanli82@163.com](mailto:xiaoyanli82@163.com)

## Supplementary Figures


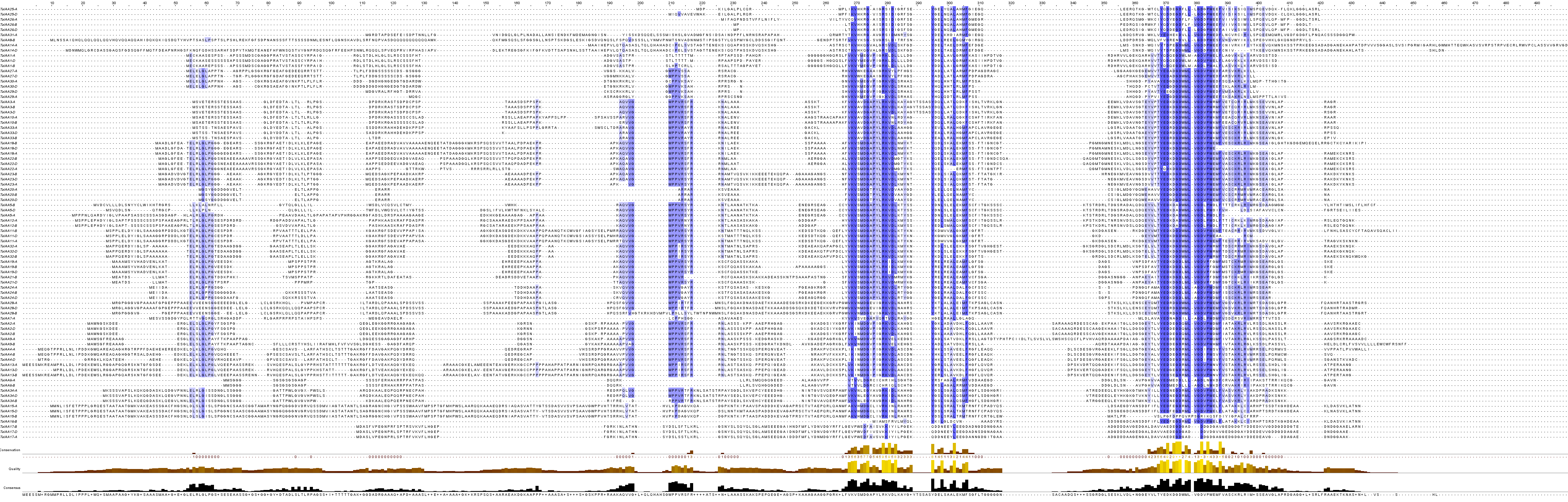

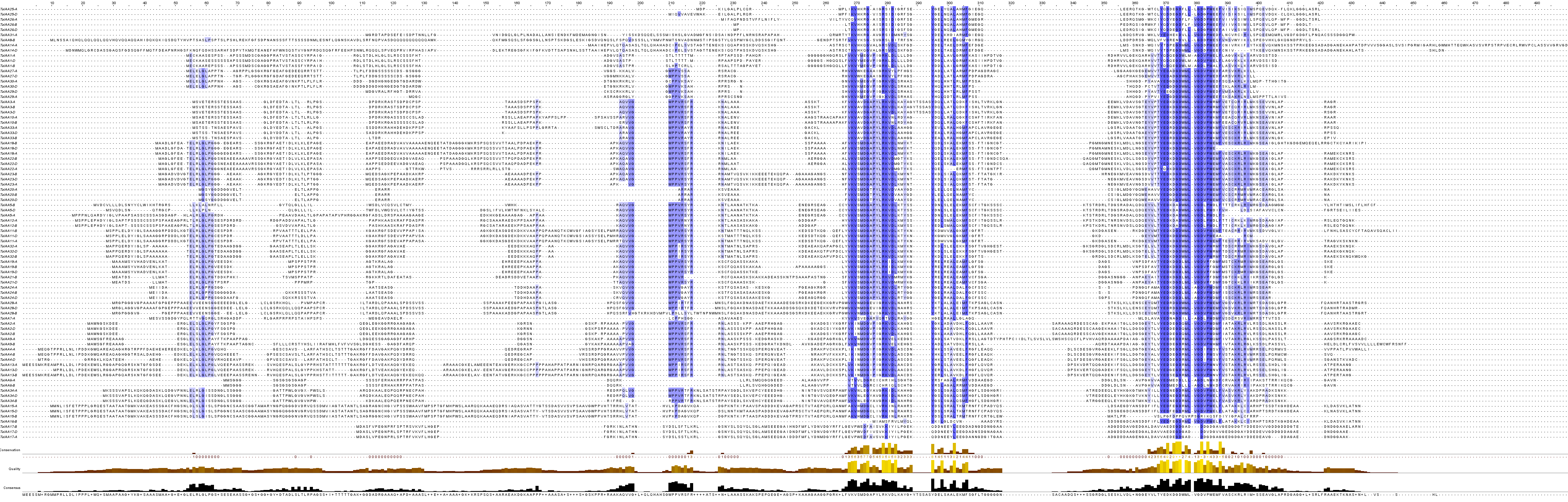


**Domain I**

**LxLxLx**

**Domain II**

**Domain III**

**NLS**

**α1**

**β**

**α2**

**Domain IV**

**NLS**

**KP**

**Supplementary Figure 1. Multiple alignment of wheat Aux/IAA proteins**. Regions I, II, III, and IV with boxes represent four conserved domains. Two NLSs and one βαα motif are showed by black rectangles and arrows, respectively. Phosphorylation sites are emphasized by triangles in the figure. Identical amino acids are highlighted by filled black columns at the bottom.

A

B

**Supplementary Figure 2. Phylogenetic relationship of *Aux/IAA* genes** between *T. urartu* and wheat A genome (A), and *Ae. tauschii* and wheat D genome (B). The branches of orthologous gene pairs were showed in red and blue, respectively

**Supplementary Figure 3. Phylogenetic relationship of Aux/IAA between barley and wheat**. The barley Aux/IAAs indicated by a red dot were used as an outgroup to calculate Ka and Ks

1. **Supplementary Tables**

**Supplementary Table 1. Genome location of *TaIAA* genes**

| Gene-A | Blast Genome Expect | Identities | LD (cM) | Gene-B | Blast Genome Expect | Identities | LD (cM) | Gene-D | Blast Genome Expect | Identities | LD (cM) |
| --- | --- | --- | --- | --- | --- | --- | --- | --- | --- | --- | --- |
| *TaIAA1-A* | 0 | 100% | 78.11 | *TaIAA1-B* | 3E-99 | 79.60% | N | *TaIAA1-D* | 0 | 100% | 55.79 |
| *TaIAA2-A* | 0 | 100% | 78.56 | *TaIAA2-B* | 0 | 100% | 64.04 | *TaIAA2-D* | 0 | 100% | 55.37 |
| *TaIAA3-A* | 0 | 100% | 78.56 | *TaIAA3-B* | 0 | 100% | 64.04 | *TaIAA3-D* | 0 | 100% | 55.37 |
| *TaIAA4-A* | 0 | 100% | 85.74 | *TaIAA4-B* | 3.00E-52 | 96.06% | N | *TaIAA4-D* | 0 | 99.67% | N |
| *TaIAA5-A* | 0 | 99.23% | N | *TaIAA5-B* | 0 | 100% | 111.09 | *TaIAA5-D* | 0 | 99.95% | N |
| *TaIAA6-A* | 0 | 100% | 111.2 | *TaIAA6-B* | 0 | 100% | 112.2 | *N* |  |  | N |
| *TaIAA7-A* | 0 | 100% | 120.3 | *N* |  |  | N | *N* |  |  | N |
| *N* |  |  | N | *TaIAA8-B* | 0 | 100% | N | *N* |  |  | N |
| *TaIAA9-A* | 0 | 100% | 57.93 | *TaIAA9-B* | 0 | 100% | 59.68 | *N* |  |  | N |
| *TaIAA10-A* | 0 | 94.86% | N | *TaIAA10-B* | 0 | 100% | 59.68 | *N* |  |  | N |
| *TaIAA11-A* | 0 | 97.92% | N | *TaIAA11-B* | 0 | 100% | N | *TaIAA11-D* | 0 | 99.90% | N |
| *TaIAA12-A* | 0 | 100% | N | *TaIAA12-B* | 0 | 100% | N | *N* |  |  | N |
| *TaIAA13-A* | 0 | 100% | 63.16 | *TaIAA13-B* | 0 | 100% | 67.48 | *TaIAA13-D* | 0 | 88.37% | N |
| *TaIAA14-A* | 0 | 92.72% | N | *N* |  |  | N | *TaIAA14-D* | 0 | 100% | 114.8 |
| *TaIAA15-A* | 0 | 100% | 25.685 | *TaIAA15-B* | 0 | 100% | 64.89 | *TaIAA15-D* | 0 | 100% | 8.81 |
| *N* |  |  | N | *TaIAA16-B* | 0 | 100% | N | *N* |  |  | N |
| *TaIAA17-A* | 0 | 100% | 28.61 | *TaIAA17-B* | 0 | 100% | 65.7 | *TaIAA17-D* | 0 | 100% | 8.81 |
| *TaIAA18-A* | 0 | 96.14% | N | *TaIAA18-B* | 0 | 100% | 46.76 | *TaIAA18-D* | 0 | 100% | 36.57 |
| *TaIAA19-A* | 0 | 98.31% | N | *TaIAA19-B* | 0 | 100% | 46.76 | *TaIAA19-D* | 0 | 100% | 36.57 |
| *TaIAA20-A* | 0 | 100% | 10.56 | *TaIAA20-B* | 0 | 100% | 70.72 | *TaIAA20-D* | 0 | 100% | 93.25 |
| *N* |  |  | N | *TaIAA21-B* | 0 | 100% | 105.65 | *TaIAA21-D* | 0 | 93.01% | N |
| *TaIAA22-A* | 0 | 100% | 54.88 | *TaIAA22-B* | 0 | 100% | 105.65 | *TaIAA22-D* | 0 | 100% | 134.075 |
| *TaIAA23-A* | 0 | 100% | 54.88 | *TaIAA23-B* | 0 | 100% | 105.65 | *TaIAA23-D* | 0 | 100% | 134.97 |
| *TaIAA24-A* | 0 | 100% | 74.09 | *TaIAA24-B* | 0 | 100% | 134.18 | *TaIAA24-D* | 0 | 100% | 153.81 |
| *TaIAA25-A* | 0 | 100% | 53.81 | *N* |  |  | N | *TaIAA25-D* | 0 | 92.95% | N |
| *TaIAA26-A* | 0 | 100% | 53.81 | *TaIAA26-B* | 0 | 88.98% | N | *TaIAA26-D* | 0 | 100% | 71.6 |
| *TaIAA27-A* | 0 | 100% | 118.54 | *N* |  |  | N | *TaIAA27-D* | 0 | 100% | 125.07 |
| *TaIAA28-A* | 0 | 100% | 134.15 | *TaIAA28-B* | 4E-11 | 87.01% | N | *TaIAA28-D* | 0 | 99.28% | N |
| *TaIAA29-A* | 0 | 100% | 79.9 | *N* |  |  | N | *TaIAA29-D* | 0 | 100% | 74.1 |
| *TaIAA30-A* | 5.00E-106 | 89.76% | N | *N* |  |  | N | *TaIAA30-D* | 0 | 100% | 74.1 |
| *TaIAA31-A* | 0 | 100% | N | *N* |  |  | N | *N* |  |  | N |
| *TaIAA32-A* | 0 | 100% | 93.26 | *TaIAA32-B* | 0 | 94.74% | N | *TaIAA32-D* | 0 | 90.90% | N |
| *TaIAA33-A* | 0 | 100% | 98.92 | *TaIAA33-B* | 0 | 100% | 82.2 | *TaIAA33-D* | 0 | 100% | 111.08 |
| *TaIAA34-A* | 0 | 94.17% | N | *TaIAA34-B* | 1E-12 | 85.71% | N | *TaIAA34-D* | 0 | 100% | 111.575 |

**Supplementary Table 2. A complete list of Aux/IAA protein sequences of wheat identified in the present study**

| Protein | Peptide length | Molecular weight | Isoelectric point | Instability index | Aliphatic index | GRAVY |
| --- | --- | --- | --- | --- | --- | --- |
| TaIAA1-A | 208 | 21859.74 | 8.48 | 47.89 | 74.93 | -0.261 |
| TaIAA1-B | 209 | 22116.2 | 7.84 | 44.39 | 86.83 | -0.106 |
| TaIAA1-D | 207 | 21769.56 | 6.68 | 50.84 | 77.67 | -0.202 |
| TaIAA2-A | 233 | 24189.22 | 9.48 | 41.52 | 70.82 | -0.461 |
| TaIAA2-B | 235 | 24290.35 | 9.36 | 43.33 | 71.07 | -0.407 |
| TaIAA2-D | 236 | 24561.64 | 8.87 | 44.93 | 70.34 | -0.44 |
| TaIAA3-A | 199 | 21508.28 | 7.04 | 46.3 | 71.46 | -0.419 |
| TaIAA3-B | 199 | 21508.28 | 7.04 | 46.3 | 71.46 | -0.419 |
| TaIAA3-D | 199 | 21524.27 | 7.04 | 48.52 | 70.96 | -0.432 |
| TaIAA4-A | 301 | 33215.55 | 6.99 | 50.81 | 73.77 | -0.61 |
| TaIAA4-B | 292 | 32155.17 | 6.52 | 51.31 | 69.04 | -0.665 |
| TaIAA4-D | 277 | 30354.25 | 7.69 | 53.95 | 69.24 | -0.636 |
| TaIAA5-A | 242 | 25182.16 | 6.01 | 51.78 | 67.05 | -0.42 |
| TaIAA5-B | 231 | 26341.75 | 8.31 | 36.29 | 91.57 | -0.048 |
| TaIAA5-D | 286 | 25163.97 | 5.43 | 38.22 | 94.87 | -0.018 |
| TaIAA6-A | 168 | 17718 | 7.82 | 52.24 | 73.65 | -0.387 |
| TaIAA6-B | 168 | 17683.98 | 7.78 | 52.58 | 77.72 | -0.35 |
| TaIAA7-A | 159 | 16806.94 | 6.75 | 38.36 | 77.85 | -0.341 |
| TaIAA8-B | 328 | 36350.96 | 4.89 | 58.09 | 66.73 | -0.723 |
| TaIAA9-A | 263 | 27300.79 | 7.27 | 37.38 | 74.66 | -0.244 |
| TaIAA9-B | 242 | 29395.62 | 7.81 | 35.01 | 85.16 | -0.028 |
| TaIAA10-A | 219 | 23630.87 | 8.32 | 63.07 | 72.61 | -0.342 |
| TaIAA10-B | 203 | 22074.98 | 8.26 | 50.73 | 71.58 | -0.427 |
| TaIAA11-A | 266 | 28822.8 | 9.09 | 42.13 | 62.57 | -0.657 |
| TaIAA11-B | 279 | 30378.85 | 8.18 | 45.17 | 71.94 | -0.379 |
| TaIAA11-D | 230 | 24842.29 | 7.79 | 44.06 | 64.76 | -0.552 |
| TaIAA12-A | 262 | 28002.58 | 8.62 | 61.82 | 64.1 | -0.612 |
| TaIAA12-B | 257 | 27398.98 | 8.82 | 60.76 | 66.84 | -0.52 |
| TaIAA13-A | 342 | 36759.43 | 6.86 | 48.75 | 69.33 | -0.646 |
| TaIAA13-B | 344 | 37213.99 | 8.05 | 52.46 | 70.32 | -0.671 |
| TaIAA13-D | 326 | 34993.6 | 8.49 | 45.86 | 73.63 | -0.608 |
| TaIAA14-A | 248 | 26984.27 | 5.74 | 49.98 | 69.07 | -0.486 |
| TaIAA14-D | 333 | 37063.51 | 5.21 | 44.04 | 49.4 | -0.848 |
| TaIAA15-A | 362 | 37878.61 | 6.85 | 42.19 | 68.53 | -0.239 |
| TaIAA15-B | 328 | 34436.18 | 9.62 | 51.85 | 66.94 | -0.237 |
| TaIAA15-D | 361 | 37627.28 | 7.08 | 45.58 | 66.28 | -0.239 |
| TaIAA16-B | 104 | 11474.99 | 4.53 | 20.13 | 81.55 | -0.192 |
| TaIAA17-A | 201 | 21173.39 | 3.57 | 30.36 | 63.05 | -0.689 |
| TaIAA17-B | 209 | 22319.51 | 3.59 | 35.74 | 62.88 | -0.74 |
| TaIAA17-D | 204 | 21644.89 | 3.48 | 27.58 | 69.7 | -0.593 |
| TaIAA18-A | 254 | 27043.79 | 5.16 | 53.96 | 67.63 | -0.417 |
| TaIAA18-B | 270 | 28914.94 | 5.19 | 54.92 | 68.66 | -0.44 |
| TaIAA18-D | 221 | 23349.3 | 4.63 | 55.01 | 68 | -0.42 |
| TaIAA19-A | 187 | 20287.38 | 8.92 | 50.96 | 64.09 | -0.446 |
| TaIAA19-B | 198 | 21083.23 | 8.92 | 51.26 | 64.06 | -0.373 |
| TaIAA19-D | 187 | 20375.44 | 8.68 | 50.96 | 63.01 | -0.488 |
| TaIAA20-A | 134 | 14731.88 | 4.76 | 36.57 | 72.63 | -0.182 |
| TaIAA20-B | 134 | 14731.88 | 4.76 | 36.57 | 72.63 | -0.182 |
| TaIAA20-D | 134 | 14761.9 | 4.76 | 37.2 | 72.63 | -0.185 |
| TaIAA21-B | 213 | 22520.55 | 7.81 | 37.02 | 61.37 | -0.454 |
| TaIAA21-D | 160 | 17167.84 | 9.08 | 36.18 | 62.64 | -0.337 |
| TaIAA22-A | 215 | 23447.01 | 9.63 | 53.84 | 71.17 | -0.368 |
| TaIAA22-B | 254 | 26892.45 | 4.99 | 54.47 | 67.23 | -0.432 |
| TaIAA22-D | 131 | 26840.39 | 4.84 | 51.61 | 67.9 | -0.416 |
| TaIAA23-A | 231 | 24640.04 | 7.99 | 42.75 | 64.61 | -0.557 |
| TaIAA23-B | 240 | 25620.22 | 9.19 | 44.17 | 64.23 | -0.598 |
| TaIAA23-D | 233 | 24802.19 | 7.98 | 42.27 | 64.05 | -0.571 |
| TaIAA24-A | 161 | 17009.15 | 5.94 | 40.38 | 62.94 | -0.398 |
| TaIAA24-B | 178 | 18637.95 | 7.83 | 45.83 | 62.99 | -0.405 |
| TaIAA24-D | 182 | 18917.19 | 5.94 | 46.52 | 61.1 | -0.336 |
| TaIAA25-A | 117 | 13067.16 | 5.31 | 46.7 | 95.86 | -0.203 |
| TaIAA25-D | 126 | 14044.38 | 5.77 | 52.17 | 104.48 | -0.024 |
| TaIAA26-A | 123 | 14138.31 | 4.48 | 48.81 | 92.62 | 0.072 |
| TaIAA26-B | 99 | 11166.73 | 4.54 | 56.62 | 81.53 | -0.208 |
| TaIAA26-D | 101 | 11184.81 | 4.54 | 51.06 | 90.7 | -0.132 |
| TaIAA27-A | 176 | 18330.65 | 5.82 | 51.27 | 68.06 | -0.33 |
| TaIAA27-D | 173 | 18228.59 | 6.6 | 54.33 | 66.4 | -0.368 |
| TaIAA28-A | 284 | 29900.7 | 6.08 | 50.94 | 65.27 | -0.494 |
| TaIAA28-B | 295 | 31323.34 | 7.32 | 48.36 | 67.79 | -0.534 |
| TaIAA28-D | 283 | 29982.76 | 5.39 | 49.31 | 67.59 | -0.488 |
| TaIAA29-A | 120 | 12917.78 | 8.88 | 59.99 | 70.42 | -0.436 |
| TaIAA29-D | 122 | 13853.06 | 10.02 | 53.92 | 74.79 | -0.527 |
| TaIAA30-A | 172 | 18667.87 | 7.12 | 39.66 | 62.16 | -0.756 |
| TaIAA30-D | 161 | 17845.04 | 6.6 | 43.8 | 65.19 | -0.803 |
| TaIAA31-A | 229 | 25264.18 | 4.44 | 43.19 | 73.16 | -0.525 |
| TaIAA32-A | 240 | 25657.23 | 8.47 | 42.63 | 66.69 | -0.581 |
| TaIAA32-B | 236 | 25461.07 | 8.47 | 43.53 | 67.83 | -0.585 |
| TaIAA32-D | 241 | 25814.4 | 8.19 | 40.67 | 67.21 | -0.583 |
| TaIAA33-A | 207 | 22410.43 | 7.81 | 54.7 | 74.9 | -0.402 |
| TaIAA33-B | 164 | 17583.18 | 6.38 | 42.65 | 87.42 | -0.122 |
| TaIAA33-D | 181 | 19476.05 | 6.85 | 46.6 | 74.89 | -0.422 |
| TaIAA34-A | 252 | 2763.96 | 7.96 | 43.84 | 69.16 | -0.734 |
| TaIAA34-B | 245 | 26992.27 | 7.5 | 42.34 | 71.15 | -0.711 |
| TaIAA34-D | 253 | 27674.98 | 6.7 | 44.4 | 71.94 | -0.68 |

**Supplementary Table 3. Information of Aux/IAA protein used in phylogenetic analysis (Figure 5)**

| Gene | Genbank ID | Function | References |
| --- | --- | --- | --- |
| *AtIAA1* | At4g14560 | Development of inflorecence and leaf | Park et al.,2002; Yang et al.,2004; Ku et al.,2008； Wasano et al.,2013 |
| *AtIAA2* | At3g23030 | Development of leaf and root | Rusak et al.,2010 |
| *AtIAA3* | At1g04240 | Elongation of cotyledon and hypocotyl, Root development, Photoperiod | Tian et al.,1999; 2002; 2014; Tatsuaki et al.,2012 |
| *AtIAA4* | At5g43700 |  |  |
| *AtIAA5* | At1g15580 | Root development | Wasano et al.,2013 |
| *AtIAA6* | At1g52830 |  |  |
| *AtIAA7* | At3g23050 | Root development, Apical dominance, Flowering, Plant height | Nagpal et al.,2000; Muto et al.,2007; Mai et al.,2011 |
| *AtIAA8* | At2g22670 | Root development | Arase et al.,2012 |
| *AtIAA9* | At5g65670 |  |  |
| *AtIAA10* | At1g04100 |  |  |
| *AtIAA11* | At4g28640 |  |  |
| *AtIAA12* | At1g04550 | Development of root and embryo | Hamann et al.,2002; Goh et al.,2012 |
| *AtIAA13* | At2g33310 | Development of root | Goh et al.,2012 |
| *AtIAA14* | At4g14550 | Development of cotyledon primordia and root, Heavy metal stress response, Plant height | Fukaki et al.,2002;2005;  Vanneste et al.,2005; Muto et al.,2007; De Smet et al.,2010; Nathan et al.,2015; Jose et al.,2015 |
| *AtIAA15* | At1g80390 | Development of root | Yan et al.,2013 |
| *AtIAA16* | At3g04730 | Development of root, ABA response | Mauro et al.,2012 |
| *AtIAA17* | At1g04250 | Gravitropism, Apical dominance, Synthesis and degradation of cell wall, Root development under salt stress | Rause et al.,1998; Worley et al.,2000; Ouellet et al.,2001; Overvoorde et al.,2005; Liu et al.,2015 |
| *AtIAA18* | At1g51950 | Development of root | Lucas et al.,2012 |
| *AtIAA19* | At3g15540 | Development of cotyledon and root, Plant height | Tatematsu et al.,2004; Saito et al.,2007; Muto et al.,2007 |
| *AtIAA20* | At2g46990 |  |  |
| *AtIAA26* | At3g16500 | Resistance to tobacco mosaic virus | Meenu et al.,2005;2006 |
| *AtIAA27* | At4g29080 |  |  |
| *AtIAA28* | At5g25890 | Development of lateral root | Rogg et al.,2001; Lucas et al.,2012 |
| *AtIAA29* | At4g32280 | Leaf senescence | Jiang et al.,2014 |
| *AtIAA30* | At3g62100 |  |  |
| *AtIAA31* | At3g17600 |  |  |
| *AtIAA32* | At2g01200 |  |  |
| *AtIAA33* | At5g57420 |  |  |
| *AtIAA34* | At1g15050 |  |  |
| *TaIAA1* | CAE00638 | Response to auxin, brassinosteroid and light | Bhumica et al.,2006 |
| *PpIAA1* | AGG19163 | Development of fruit | Shi et al.,2014 |
| *VvIAA4* | AEV89794 | Stress response, Development of root,leaf and inflorecence | Birsen et al.,2013 |
| *VvIAA9* | ADR80320 | Development of leaf, Fruit set | Keiko et al.,2012; Jung et al.,2014 |
| *ZmIAA10* | B6TTY7 | Development of root | Woll et al.,2005; von Behrens et al.,2011 |
| *ZmIAA23* | K7ULU8 | Development of root | Yvonne et al.,2013 |
| *SbIAA1* | EER90734 | Stress tolerance (induced by IAA, BR, Salt and Drought) | Wang et al.,2010 |
| *SlIAA1* | JN379347 | Development of root |  |
| *SlIAA3* | NP_001266029 | Development of root | Chaabouni et al.,2009 |
| *SlIAA9* | AEX00353 | Leaf morphogenesis, Fruit set, Root development | Wang et al.,2005; Andrea et al.,2015 |
| *SlIAA14* | AEX00357 | Root development, Hormone response | Li et al.,2010 |
| *SlIAA15* | AEX00358 | Development of fruit | Deng et al.,2012 |
| *SlIAA17* | AEX00360 | Development of fruit | Su et al.,2014 |
| *SlIAA27* | AEX00366 | Development of fruit and root | Deng et al.,2012; Carole et al.,2012;2013 |
| *CsIAA2* | BAA85821 |  |  |
| *OsIAA1* | Os1g08320 | Plant type, Root length | Thakur et al.,2001; Zhang et al.,2008; Song et al.,2009 |
| *OsIAA2* | Os1g09450 | Enhance the resistance to pathogen | Chen et al.,2009 |
| *OsIAA3* | Os1g13030 | Formation of root and leaf | Nakamura et al.,2006; Liu et al.,2015 |
| *OsIAA4* | Os1g18360 | Tiller angle, Plant height | Song et al.,2009;2013 |
| *OsIAA5* | Os1g48450 | Highly expressed in roots, Drought resistance, Yield-related | Jain et al.,2006; Zvi et al.,2011 |
| *OsIAA6* | Os1g53880 | Drought resistance, Enhance the resistance to pathogen, Tiller outgrowth, Highly expressed in roots | Jain et al.,2006; Chen et al.,2009; Jung et al.,2015 |
| *OsIAA7* | Os2g13520 |  |  |
| *OsIAA8* | Os2g49160 |  |  |
| *OsIAA9* | Os2g56120 | Highly expressed | Mukesh et al.,2006 |
| *OsIAA10* | Os2g57250 |  |  |
| *OsIAA11* | Os3g43400 | Formation of lateral root | Zhu et al.,2012; Liu et al.,2013; Zhang et al.,2014 |
| *OsIAA12* | Os3g43410 |  |  |
| *OsIAA13* | Os3g53150 | Formation of lateral root | Yuka et al.,2012 |
| *OsIAA14* | Os3g58350 | Highly expressed | Mukesh et al.,2006 |
| *OsIAA15* | Os5g08570 |  |  |
| *OsIAA16* | Os5g09480 |  |  |
| *OsIAA17* | Os5g14180 |  |  |
| *OsIAA18* | Os5g44810 | ABA response, Root development. | Zhao et al.,2015 |
| *OsIAA19* | Os5g48590 | Highly expressed | Mukesh et al.,2006 |
| *OsIAA20* | Os6g07040 | Highly expressed | Mukesh et al.,2006; Arite et al.,2007 |
| *OsIAA21* | Os6g22870 | Drought resistance, Yield-related | Zvi et al.,2011 |
| *OsIAA22* | Os6g24850 |  |  |
| *OsIAA23* | Os6g39590 | Root development | Jain et al.,2006; Ni et al.,2014； |
| *OsIAA24* | Os7g08460 | Highly expressed | Mukesh et al.,2006 |
| *OsIAA25* | Os8g01780 |  |  |
| *OsIAA26* | Os9g35870 |  |  |
| *OsIAA27* | Os11g11410 |  |  |
| *OsIAA28* | Os11g11420 |  |  |
| *OsIAA29* | Os11g11430 |  |  |
| *OsIAA30* | Os12g40890 |  |  |
| *OsIAA31* | Os12g40900 | Highly expressed | Mukesh et al.,2006 |

**Supplementary Table 4. Plex probes and expression data of *TaIAA* genes in nineteen organs in wheat**

| **Qry ID** | **Tgt ID** | **Co** | **Me** | **SL** | **SA** | **SR** | **Sh** | **FL** | **Le** | **Sh** | **Ro** | **In** | **Sp** | **Spi** | **An** | **Pi** | **Gl** | **Ca** | **Em** | **En** |
| --- | --- | --- | --- | --- | --- | --- | --- | --- | --- | --- | --- | --- | --- | --- | --- | --- | --- | --- | --- | --- |
| *TaIAA1* | Ta.3163.1.S1_at | -1.315 | -1.015 | -0.897 | -1.574 | -1.206 | -1.703 | -0.510 | -0.304 | -1.344 | -1.635 | -1.447 | -1.491 | -1.604 | -1.627 | -1.491 | -0.980 | -1.403 | -0.634 | -1.128 |
| *TaIAA2,9* | TaAffx.31689.1.S1_at | -0.884 | 0.682 | -0.254 | 0.475 | -0.503 | 0.587 | -1.499 | -0.802 | -0.273 | -0.932 | 0.209 | -0.925 | -0.688 | -2.114 | -0.782 | -1.985 | -1.890 | -1.264 | -1.199 |
| *TaIAA3* | Ta.10032.1.S1_a_at | 0.979 | 1.466 | 1.280 | 0.990 | 1.747 | 1.126 | 0.942 | 0.947 | 1.530 | 1.549 | 1.666 | 1.053 | 0.958 | 0.665 | 1.814 | 1.016 | 1.386 | 1.431 | 1.058 |
| *TaIAA4* | Ta.9390.2.S1_at | -0.043 | -0.458 | -0.387 | -0.891 | 0.676 | -0.904 | -0.595 | -0.688 | -0.967 | -0.086 | -0.317 | -0.654 | -0.836 | -0.235 | -0.877 | -0.105 | -1.969 | -1.185 | -1.688 |
| *TaIAA5* | Ta.674.1.S1_at | 1.224 | 1.048 | 0.576 | 0.786 | 1.416 | 0.963 | 0.383 | 0.232 | 1.366 | 1.162 | 1.214 | 0.514 | 0.582 | 0.320 | 0.671 | 0.953 | -0.074 | 1.116 | -1.078 |
| *TaIAA6* | Ta.19375.1.S1_a_at | -1.128 | -0.960 | -0.445 | -1.688 | 1.851 | -1.149 | -1.582 | -0.105 | -1.719 | 2.489 | -0.967 | 0.899 | 0.599 | -1.156 | 0.226 | 1.326 | -0.355 | -1.559 | 0.302 |
| *TaIAA7,24* | TaAffx.78729.1.S1_x_at | -0.497 | -0.973 | -0.555 | -0.654 | -1.235 | -0.311 | -0.266 | -0.387 | 0.279 | -1.286 | -0.795 | -0.918 | -0.406 | -0.654 | 0.042 | -1.178 | -1.071 | -0.273 | -1.071 |
| *TaIAA8* | TaAffx.78729.1.S1_at | 1.446 | 0.824 | -2.262 | 1.564 | -0.497 | 1.401 | -2.329 | -2.796 | 1.079 | -0.960 | 1.315 | 0.126 | 0.737 | -0.154 | -1.171 | -1.529 | -0.960 | 0.331 | -3.344 |
| *TaIAA10* | Ta.9550.1.A1_at | -0.575 | -0.582 | -0.292 | -0.497 | -0.080 | -0.555 | -0.235 | -0.355 | -0.451 | -0.311 | -0.608 | -0.768 | -0.595 | -0.615 | -0.336 | -1.008 | -0.575 | -0.795 | -0.967 |
| *TaIAA11* | TaAffx.121900.1.S1_at | 1.280 | 1.280 | 1.219 | 1.656 | 1.485 | 1.646 | 1.074 | 2.742 | 1.011 | 1.167 | 1.675 | 1.426 | 1.515 | 0.446 | 1.671 | 1.431 | 0.770 | 1.300 | 0.509 |
| *TaIAA12* | TaAffx.105950.1.S1_x_at | 0.671 | 0.791 | 0.813 | 1.084 | 1.214 | 1.069 | 0.802 | 0.709 | 0.565 | 1.000 | 1.048 | 0.383 | 0.554 | -0.154 | 0.654 | 0.660 | -0.056 | 0.942 | 0.048 |
| *TaIAA14* | TaAffx.117504.1.S1_at | 2.032 | 2.288 | 2.217 | 2.297 | 2.186 | 2.332 | 2.359 | 2.279 | 2.041 | 2.257 | 2.783 | 2.109 | 2.195 | 2.385 | 2.763 | 2.557 | 2.055 | 2.557 | 2.288 |
| *TaIAA13* | Ta.2593.2.S1_at | -1.135 | 0.261 | 1.280 | 0.179 | 0.682 | 0.360 | -0.062 | 0.693 | 0.096 | 0.759 | -0.621 | -0.721 | -0.688 | -0.019 | -2.017 | -0.304 | -0.536 | -0.374 | 0.372 |
| *TaIAA16* | Ta.10395.1.S1_a_at | -0.714 | -0.368 | -0.516 | -0.822 | -0.728 | -0.582 | -0.285 | -0.361 | -0.523 | -1.337 | 0.372 | -0.575 | -0.884 | -0.129 | -1.264 | -1.344 | -0.822 | -0.191 | -0.994 |
| *TaIAA15* | TaAffx.27045.1.S1_at | -1.022 | 0.072 | 0.905 | -0.311 | 0.676 | 0.054 | -0.393 | 0.429 | 0.011 | 0.660 | -0.782 | -0.856 | -0.641 | -0.086 | -1.985 | -0.381 | -0.529 | -0.628 | 0.232 |
| *TaIAA17* | Ta.10395.1.S1_x_at | -0.918 | -1.078 | -1.300 | -1.015 | -1.447 | -0.918 | -0.960 | -0.911 | -1.022 | -1.322 | -0.946 | -1.015 | -1.242 | -0.674 | -0.877 | -1.604 | -0.891 | -0.987 | -1.107 |
| *TaIAA18,22,*  *33,34* | TaAffx.11969.1.A1_at | 2.402 | 2.235 | 1.341 | 2.014 | 2.096 | 2.041 | 0.531 | 0.167 | 2.172 | 2.132 | 2.195 | 1.011 | 1.723 | 1.126 | 2.463 | 0.514 | 0.883 | 1.912 | 0.250 |
| *TaIAA19* | Ta.1890.1.S1_x_at | 1.090 | 0.791 | -0.870 | 0.214 | 0.682 | 0.759 | -1.750 | -1.890 | 0.856 | 0.576 | -0.260 | -1.257 | -0.748 | -2.559 | 0.824 | -1.843 | -1.727 | 0.232 | -3.079 |
| *TaIAA20,27* | Ta.23069.2.S1_a_at | -1.135 | -1.114 | -0.782 | -0.939 | 0.463 | -1.597 | -1.199 | -1.185 | -0.856 | 0.138 | -1.432 | -1.286 | -0.904 | -1.249 | -1.574 | -1.469 | -0.755 | -0.932 | -1.403 |
| *TaIAA23* | Ta.25219.1.A1_at | 2.306 | 2.177 | 0.958 | 1.766 | 2.367 | 1.738 | 0.372 | 0.161 | 1.912 | 2.118 | 2.078 | 1.178 | 1.244 | 0.273 | 2.574 | 1.209 | 0.531 | 1.675 | 0.042 |
| *TaIAA21* | Ta.4938.1.S1_at | 0.905 | 0.931 | -1.121 | 0.167 | 1.603 | 0.320 | -1.827 | -2.354 | 0.671 | 1.157 | -0.471 | -0.523 | -0.323 | -2.490 | 1.823 | -0.043 | -1.380 | 0.604 | -1.938 |
| *TaIAA26* | Ta.7905.1.S1_a_at | -1.820 | 0.308 | -1.022 | 0.179 | -2.147 | 0.537 | 0.132 | -0.298 | -0.432 | -1.092 | 0.660 | 0.937 | 0.846 | -0.062 | 1.090 | -0.361 | 0.024 | -0.568 | 0.232 |
| *TaIAA25* | Ta.6746.1.S1_at | -1.015 | 0.179 | -0.254 | 0.197 | -0.628 | 0.325 | 0.435 | 0.078 | -0.311 | -0.092 | 0.526 | 1.162 | 0.764 | -0.304 | 1.203 | 1.300 | 0.395 | -0.279 | 0.492 |
| *TaIAA28* | Ta.28513.1.S1_s_at | -0.829 | -0.648 | -0.037 | -0.523 | -0.484 | -0.406 | 0.514 | 0.203 | -0.204 | -0.795 | -0.043 | -0.361 | -0.654 | -1.001 | -1.358 | -0.135 | -0.741 | -0.575 | -1.036 |
| *TaIAA29,30* | TaAffx.27045.3.S1_s_at | -2.279 | -2.221 | -2.823 | -2.968 | -0.870 | -3.239 | -3.154 | -3.154 | -1.938 | -1.015 | -3.014 | -3.060 | -2.346 | -2.430 | -2.977 | -3.125 | -2.620 | -1.985 | -2.559 |
| *TaIAA31* | Ta.16912.1.S1_at | -3.239 | -3.042 | -2.778 | -2.950 | -2.823 | -3.421 | -2.524 | -2.752 | -1.898 | -2.699 | -3.014 | -3.191 | -3.107 | -2.337 | -3.042 | -2.941 | 0.526 | 1.406 | 0.576 |
| *TaIAA32* | TaAffx.12089.1.S1_at | 0.366 | 0.969 | 0.726 | 1.446 | 0.423 | 1.198 | -0.266 | -0.031 | 0.974 | 0.520 | 1.680 | 0.764 | 0.867 | 1.346 | 1.968 | 0.786 | 0.042 | 0.899 | -0.160 |
| *TaIAA7* | Ta.3164.2.S1_at | -0.523 | -0.939 | -0.575 | -0.555 | -1.171 | -0.342 | -0.285 | -0.342 | -0.191 | -0.994 | -0.829 | -0.588 | -0.555 | -0.582 | -0.086 | -0.555 | -0.939 | -0.721 | -1.036 |

The organs of wheat were as follows: Co (coleoptile), Me (mesocotyl), SL (seeding leaf), SA (shoot apex), SR (seeding root), Sh (shoot), FL (flag leaf), Le (leaf), Sh (sheath), (roots), In (inflorecence), Sp (spike), Spi (spikelet), An (anther), Pi (pistil), Gl (glume), Ca (caryopsis), Em (embryo), and En (endosperm).

**Supplementary Table 5. Orthologous gene pairs and chromosomal location of *TuIAA* and *AetIAA* genes**

| *TaIAA-A* | *TuIAA* | Tu Chro. |  | *TaIAA-D* | *AetIAA* | Aet Chro. | LD Map (cM) |
| --- | --- | --- | --- | --- | --- | --- | --- |
| *TaIAA1A* | *TRIUR_21365* | 1AS1-0.47-0.86 |  | *TaIAA1-D* | *AEGTA00185* | 1DS | 49.6 |
| *TaIAA3A* | *TRIUR_34968* | 1AS1-0.47-0.86 |  | *TaIAA3-D* | *AEGTA27665* | 1DS | 54.01 |
| *TaIAA9A* | *TRIUR_31949* | 3AS4-0.45-1.00 |  | *TaIAA2-D* | *AEGTA14269* | 1DS | 54.97 |
| *TaIAA10A* | *TRIUR_08262* | 3AS4-0.45-1.00 |  | *TaIAA4-D* | *AEGTA36436* | 1DL | 97.75 |
| *TaIAA11A* | *TRIUR_25659* | 3AS4-0.45-1.00 |  | *TaIAA4-D* | *AEGTA22431* | 1DL | 97.75 |
| *TaIAA27A* | *TRIUR_15460* | 3AS2-0.23-0.45 |  | *TaIAA13-D* | *AEGTA32199* | 3DS | 76.51 |
| *TaIAA32A* | *TRIUR_27590* | C-3AL3-0.42 |  | *TaIAA11-D* | *AEGTA27928* | 3DS | 76.97 |
| *TaIAA13A* | *TRIUR_24631* | 3AL3-0.42-0.78 |  | *TaIAA5-D* | *AEGTA15903* | 3DL | 123.12 |
| *TaIAA15A* | *TRIUR_18016* | 4AL12-0.43-0.59 |  | *TaIAA17-D* | *AEGTA05621* | 4DS | 55.48 |
| *TaIAA18A* | *TRIUR_13985* | 5AS3-0.75-0.98 |  | *TaIAA18-D* | *AEGTA26128* | 5DS | 37.38 |
| *TaIAA22A* | *TRIUR_01367* | 5AL10-0.57-0.78 |  | *TaIAA20-D* | *AEGTA22565* | 5DL | 89.93 |
| *TaIAA23A* | *TRIUR_17789* | 5AL10-0.57-0.78 |  | *TaIAA21-D* | *AEGTA30995* | 5DL | 121.14 |
| *TaIAA24A* | *TRIUR_13609* | 5AL17-0.78-0.87 |  | *TaIAA23-D* | *AEGTA29089* | 5DL | 123.7 |
| *TaIAA28A* | *TRIUR_01945* | 6AL8-0.90-1.00 |  | *TaIAA24-D* | *AEGTA30959* | 5DL | 151.96 |
| *TaIAA30A* | *TRIUR_08921* | 7AS8-0.45-0.59 |  | *TaIAA22-D* | *AEGTA35747* | 5DL | 184.36 |
| *TaIAA33A* | *TRIUR_02488* | 7AL1-0.39-0.71 |  | *TaIAA28-D* | *AEGTA13779* | 6DL | 142.91 |
|  |  |  |  | *TaIAA25-D* | *AEGTA32434* | 6DL | 157.38 |
|  |  |  |  | *TaIAA26-D* | *AEGTA09874* | 6DL | 157.38 |
|  |  |  |  | *TaIAA29-D* | *AEGTA22007* | 7DS | 44.37 |
|  |  |  |  | *TaIAA30-D* | *AEGTA29038* | 7DS | 112.74 |
|  |  |  |  | *TaIAA34-D* | *AEGTA02351* | 7DL | 117.25 |
|  |  |  |  | *TaIAA32-D* | *AEGTA05214* | 7DL | 118.43 |
